# Perceptions of genetic testing in patients with hereditary chronic pancreatitis and their families: a qualitative triangulation

**DOI:** 10.1038/s41431-020-00705-9

**Published:** 2020-08-12

**Authors:** Regina Müller, Ali A. Aghdassi, Judith Kruse, Markus M. Lerch, Peter Simon, Sabine Salloch

**Affiliations:** 1 Institute of Ethics and History of Medicine, University of Tübingen, Gartenstraße 47, 72074 Tübingen, Germany; 2 Department of Medicine A, University Medicine Greifswald, Ferdinand-Sauerbruch-Straße, 17475 Greifswald, Germany; 3 Institute of Ethics and History of Medicine, University Medicine Greifswald, Ellernholzstr. 1-2, 17487 Greifswald, Germany; 4 Institute of History, Ethics and Philosophy of Medicine, Hannover Medical School, Carl-Neuberg-Str. 1, 30625 Hannover, Germany

**Keywords:** Medical ethics, Quality of life, Health services, Ethics, Psychology

## Abstract

Hereditary chronic pancreatitis (HCP) is a genetically determined condition characterized by intermittent acute episodes of pancreatitis and long-term impairment of the exocrine and endocrine pancreatic functions. Genetic test results can have substantial psychological and social consequences for the individuals tested and their families. Nevertheless, little is known so far about the subjective experience of individuals genetically tested for HCP. This qualitative study examines the viewpoints of HCP patients and their relatives in order to identify the psychosocial and ethical implications related to genetic testing within families. Semi-structured qualitative individual interviews and a focus group with HCP patients and their family members were conducted. Data were audio-recorded, transcribed verbatim and analysed using qualitative content analysis. A total of 28 individuals were enrolled in the study: 24 individuals (17 patients, 7 relatives) were interviewed in semi-structured one-on-one interviews and 4 individuals (2 patients, 2 life partners) participated in the focus group. Emerging topics covered (1) genetic testing in childhood, (2) genetic testing within the family and (3) family planning. The study reveals that genetic testing for HCP has a wide influence in familial contexts and is accompanied by normative issues, such as autonomy, reproductive decisions and sharing of information within the family. The results raise the awareness of the complexity of family contexts: familial relationships and dynamics can have great influence on the individual decisions related to genetic testing. Increased understanding of these relational contexts can help health professionals, for example, in counselling, to discuss genetic testing better with patients and families.

## Introduction

Hereditary chronic pancreatitis (HCP) is a rare variety of chronic pancreatitis (CP) which is characterized by intermittent acute episodes of pancreatitis and long-term impairment of the exocrine and endocrine pancreatic functions [1] due to loss of parenchymal tissue and formation of fibrosis [2]. The term ‘hereditary pancreatitis’ is usually reserved for a category of the disease associated with germline mutations in the cationic (PRSS1) trypsinogen gene [3] and distinguished from other varieties, which can also be associated with genetic risk factors but are not inherited in an autosomal dominant manner [4]. The latter are sometimes referred to as familial pancreatitis.

The clinical presentation can include recurrent abdominal pain, nausea and vomiting, maldigestion, pseudocyst formation [5], and bile duct [6] and duodenal obstruction [7]. Currently, there is no causative treatment for HCP and therapy focuses, as in other forms of CP [8], on pain management, therapy for endocrine and exocrine insufficiency, and endoscopic or surgical interventions for complications [9]. The course of the disease varies from asymptomatic to very severe forms [10].

As a rare genetic disorder, HCP is diagnosed predominantly in individuals of European origin [11]. It was first described as a genetically determined disease in 1952 [12] and later identified to be associated with mutations in the cationic trypsinogen (PRSS1) gene [13]. It required extensive experimental research [14] before it was discovered that the underlying mechanism involves the resistance of the disease-relevant, mutant trypsinogen isoforms against degradation by chymotrypsinogen C [15]. However, despite comprehensive research, many unanswered questions regarding HCP remain and the identification of further mutations and the interplay of genetic, epigenetic and environmental factors are the focus of current studies.

CP, in itself, represents a psychological burden for the patients affected [16]. Suffering from a genetic form of the disease carries an additional dimension for patients and their relatives. Currently, genetic testing by direct DNA sequencing is available for many diseases. It is widely discussed not only in biomedical research but also from sociological, psychological and ethical perspectives [17–20]. The complexities of dealing with genetic test results, consequences of genetic knowledge, impacts on families, discrimination and stigmatization are the focus of the debates [17–20]. Regarding families, topics such as prenatal testing, reproductive decisions, sharing of information within the family and attitudes regarding genetic testing have been discussed [21–25].

The effects of genetic information depend on many factors, such as the condition being tested and the social context of the test [26]. Regarding HCP, genetic testing can lead to early diagnosis and insofar prevent the further search for and misattribution of the underlying cause of the disease. A diagnosis of HCP also provides a causative explanation to patients about the origin of their underlying disease which may facilitate coping with the disease. It can also provide useful prognostic information and options for family planning [27, 28].

The International Association of Pancreatology (IAP) formulated criteria for genetic testing for HCP which refer to the clinical presentation, the family history and the eligibility for study participation (Table 1) [29]. In addition to these indications, the recommendation of the IAP addresses especially the counselling process and privacy issues [29]. Moreover, ethical issues, such as patient autonomy, informed consent, prenatal testing, testing in minors and the impact on family members, are debated in the recommendation of the IAP and in other contributions [20, 27].

**Table 1:** Criteria for genetic testing for HCP according to the International Association of Pancreatology [ref. 29].

Criteria for genetic testing for HCP
Patients with recurrent attacks of acute pancreatitis without explanation
Patients with idiopathic chronic pancreatitis
Individuals with a family history of pancreatitis in a first- or second-degree relative
Children with an unexplained episode of documented pancreatitis who require hospitalization and where there is significant concern that hereditary pancreatitis should be excluded
Patients with pancreatitis eligible for an ethics committee approved research protocol

However, little is known regarding the impact of such testing on patients and their family’s lives. The current study is the first qualitative study focusing on both the viewpoints of HCP patients *and* their relatives on the genetic testing for HCP. The psychosocial and ethical implications associated with HCP genetic testing are discussed not only for the individual but also for the family. The involvement of relatives in the current study can help to reach a comprehensive picture of the effects of genetic testing on family life. The study specifies the experiences with genetic testing regarding HCP but expands, at the same time, the existing research on genetic information within families. In doing so, the study aims to explore the psychosocial and ethical implications of genetic testing for HCP to elucidate the impact of genetic testing for rare chronic diseases in family contexts.

## Materials and methods

Qualitative triangulation was used to investigate how genetic testing affects patients and their relatives’ lives. Triangulation, as the combination of different approaches to study the same object of inquiry, can refer to such different aspects as data, investigators, theories or methods. Method triangulation can refer to the combination of quantitative methods, qualitative methods or both. Method triangulation in qualitative inquiry means a multimethod approach to qualitative data collection and analysis, which can refer, for example, to the combination of qualitative methods such as one-on-one interviews and focus groups [30]. The underlying idea of all approaches is to study the respective phenomenon from different perspectives in order to gain a more complete picture and deepen the understanding [30]. In the present study, individual interviews were supplemented by a group session to discuss the psychosocial and ethical aspects of genetic testing within families.

As participants can contradict or complement each other in discussions, ethical issues, which are often vague and implicit, can be well crystallized in group sessions. Throughout the discussion, different ethical dimensions of a topic can be collected, which, in turn, can strengthen the findings and enrich the interpretation. Furthermore, the focus group can be seen as a test of validity of the results from the individual interviews. The additional group session can help to reduce biases or deficiencies caused by one-on-one interviews with a researcher. In the group session, the participants talk among themselves, which can lead to a better integrity and consistency of the research findings.

The interview guide for the individual interviews was developed containing four major topics: patient biography, experience with genetic testing, biomedical research and patient self-help groups. The interview guide was pilot tested. One interview with a patient and one interview with a relative were conducted as pilots face-to-face by RM. As only minimal changes to the interview guide emerged from the pilot testing, these two interviews were included in the final analysis. The interview guide was used for individual interviews with patients and family members (Suppl. 1). Based on the results of the individual interviews, the interview guide for the focus group, targeting the topic of genetic testing within families, was developed (Suppl. 2).

The study was approved by the Ethics Committee of the University Medicine Greifswald. Written informed consent was obtained from all study participants. Research ethics requirements, such as data anonymity, were observed diligently.

### Study participants: sampling for individual interviews and focus group

The study sample was drawn from individuals participating in a German self-help organisation for patients with HCP and their families. MML and PS, who have a longstanding contact to the patient organisation, established contact with the chairperson. The latter passed on the request to participate in the study to the members of the organisation. Patients who responded to this call, volunteered to participate in the study and identified themselves as HCP patients. They were sent an e-mail invitation by RM. When the person contacted confirmed his/her interest, written information about the context and goals of the study were sent by post and RM contacted the prospective participants additionally by telephone to resolve potential questions. Participants recruited in this way were asked whether they would forward the invitation to participate in the study to further patients and relatives (snowballing technique).

Inclusion criteria restricted the sample to patients who already had HCP in their families, had been tested for the hereditary form or had thought about a genetic test. Inclusion criteria regarding family members allowed the participation of parents, children, siblings, aunts, uncles, spouses and life partners. All participants had to be at least 18 years old. The participant selection aimed for the greatest possible variation in terms of age, gender, level of education, familial status and disease progression. Sampling was discontinued when data saturation was reached. Data saturation was defined as the point when no new relevant information regarding the aim of the study emerges and the codes become repetitive with only small variations [31].

### Data collection

The individual interviews were conducted in a semi-structured style, face-to-face or via the telephone by RM (female PhD student) who has been trained in empirical bioethics and qualitative research. Field notes were made during and after the individual interviews. The interviews were audio-recorded, transcribed verbatim and pseudonymized. In addition, a focus group session with patients and life partners was carried out. Based on the analysis of the individual interviews, the main topic ‘genetic testing’ was selected for discussion in the focus group. The group session was conducted by the interviewer RM and one assistant. It was audio-recorded, transcribed verbatim and pseudonymized.

### Data analysis

The transcripts were analysed by RM and SS using qualitative content analysis according to Mayring to identify codes and categories [32], with the aid of the software program MAXQDA12. The transcripts were encoded, codes and categories were regularly discussed and modified in team meetings, and a coding scheme was developed. The coding scheme was inductively expanded and critically revised. Once theoretical saturation and redundancy had been reached, the results were further interpreted regarding the emerging categories. Rater influence was controlled in team discussions during the coding process and by researchers with different professional backgrounds (medicine, philosophy, and ethics) involved in the data interpretation (Suppl. 3).

## Results

The study was conducted between July 2017 and October 2019 in Germany. A total of 28 individuals were enrolled in the study. Two potential participants declined to be interviewed for personal reasons. Twenty-four individuals were interviewed in semi-structured individual interviews (17 patients, 7 relatives) and four individuals (2 patients, 2 life-partners) participated in the focus group.

Potential participants for the focus group were reluctant to discuss the sensitive and private issues of genetic testing in a larger group. Consequently, the focus group session was relatively small consisting of two patients and their partners. The group represented a so-called real group [33], a group that had not been composed specifically for research but existed independently of the research situation. The participants of the group discussion were already familiar with each other and had had similar experiences because of their involvement in the patient organisation.

Twenty-two of the individual interviews took place at the participants’ homes; two interviews were conducted by telephone. The focus group session was held in the context of the annual meeting of the patient organisation. The one-on-one interviews lasted an average of 44 min (median: 43 min), ranging from 16 to 91 min. The focus group took 75 min.

The study included patients in different stages of the disease. The patients had had a clinically overt disease either since their birth, childhood or adulthood. One patient was in an acute phase of the disease during the interview study. Some participants had multiple roles. One participant, for example, was the partner of a patient and, at the same time, the parent of an affected child. As a result of the multiple roles, many different but interwoven familial relationships are covered in the present study. In order to manage this complexity, each participant was formally assigned only one role. The participants themselves chose their roles, which resulted in the three categories: patient, partner and parent. Additional characteristics of the interview participants can be seen in Table 2.

**Table 2:** Sample characteristics (individual interviews).

	Patients (*n* = 17)	Relatives (*n* = 7)	Total (*n* = 24)
Age	20–70 (median: 49)	47–78 (median: 67)	20–78 (median: 52,5)
Age groups			
18–30	2		2
30–50	7	1	8
50–70	7	5	12
70–90	1	1	2
Gender			
Male	7	3	10
Female	10	4	14
Education			
A level	10	2	12
Secondary school	5	2	7
Other	2	3	5
Marital status			
Single	5		5
Married	11	7	18
Living together	1		1
Having children	12	7	19
Employment	13	5	18
Member of self-help group	11	3	14
Genetically tested	11		11
In acute episode	1		1
Relationship to patient			
Parent		3	3
Spouse		4	4

The codes identified from about the 20th interview were not novel in substance but variations on topics which existed already. Four more individual interviews were conducted to make sure that the point of data saturation had been reached. These additional one-on-one interviews confirmed that data saturation had been reached. The focus group was seen as a further validation tool in order to get a robust picture.

Genetic testing in the context of families was identified in the individual interviews as an important but complex issue, associated with different ethical questions. For this reason, the topic of genetic testing was chosen for further discussion in the group session and as a focus of the current paper. Selected study results will be presented in the following with a focus on the impact of genetic testing on patients and their family’s lives, particularly regarding (1) genetic testing in childhood, (2) genetic testing undergone by families together, and (3) family planning. Since HCP patients are a relatively small group in Germany, characteristics, such as gender and age are not mentioned in the following quotes in order to guarantee data anonymity.

### Genetic testing in childhood

The study participants debated the topic of genetic testing during childhood, referring to tests in their own childhood and tests for their children. A few participants did not remember whether a test was done during their childhood. Some participants reported that a test had been done, but that they were not informed about the test results. Other participants remembered the testing process but did not remember the test results.

*Well, I didn’t notice that it [genetic testing] was done, […] when I was twelve years old, it was just said, we had this genetic defect. [Interview 18, Patient]*

*Once [the physician] did a genetic test, but I never got an answer. [Interview 1, Patient]*

Many participants were unsure how they themselves could assess and judge genetic testing in childhood. Regarding the optimal time for testing, for example, testing at different ages and for different reasons were suggested. Participants explained that genetic testing was such a highly individual decision that the right time for testing could not be determined in general. Instead, it depended on when the first symptoms occurred and how the person affected utilized the test results. Although different ages for testing were discussed, many participants named adolescence as an appropriate time for testing. Reasons against earlier testing were that children have had too little life experience and must first develop the ability to understand and decide about this complex issue. Testing immediately after birth and in early childhood was, therefore, rejected by most of the study participants. On this point, no differences between patients and family members could be observed and consensus was also reached in the focus group. Tests in adolescence were supported by many participants because, in their view, genetic testing could lead to certainty about the disease and its origin and the testing could have a reassuring effect.

*I would say, early in [adult] life, because then it brings more certainty that you know where it comes from. [Focus group]*

*As I said, it’s very, very difficult, when you are a kid. As a kid, you are inexperienced anyway. [Focus group]*

In addition, the role and responsibility of the parents were addressed by the participants. Some found it essential that the parents know about the genetic status of the disease to be able to react appropriately. By contrast, other participants emphasized that it could have strong negative consequences for the subsequent childhood if parents panic as a reaction to the test results and put their children under strong surveillance.

*I think it’s also very important […] how the parents react at that moment. Do they panic ‘we have to do this and that’ or do they deal with it very calmly and sensibly? I think this is very important, even for the rest of your life. It shouldn’t be underestimated. Of course, taking precautions, but there are, I say, these ‘helicopter parents’: ‘Rather not, better not, not at all, and no, you aren’t allowed to go to friends and eat elsewhere,’ although there’s nothing yet. [Focus group]*

Some patients reported that their parents had been concerned about their further development as a consequence of the test results and that they, therefore, had been taught to be cautious about various aspects of life. Parents had restricted, for example, physical activities, such as sports, leisure activities, such as horse riding, or going abroad. One participant reported that he/she had been excluded from sports classes in his/her childhood due to HCP, although—from his/her own perspective—he/she would have been able to do sports. In this context, the participants also reflected on their own biography with the disease and the complex interaction of disease, environment and their own behaviour.

*But I also know that it [the disease] is not the only factor […]. Of course, I don’t know, my childhood itself, living with this disease: what made me what I’m today? Not everything can be attributed to the condition, but also the circumstances that I had, how they changed me, my personality, my character. I think it all comes together. [Interview 19, Patient]*

### Genetic testing within the family

Participants also reported how entire families had undergone genetic testing together, but that the issue had not been discussed previously within the family. Several participants (belonging to one family), for example, described that an appointment had been made for them all to go to the physician together and undergo the test one by one. Some said that the question of whether to undergo the test together had been a simple question of ‘yes or no’. Others reported that the question had not been asked at all.

*Why should you make huge discussions about this? Either Yes or No. [Interview 10, Relative]*

*No, this wasn’t really discussed much, because it was always clear that I would get maximum support, so to say. So, it was clear, okay, we are here together now, we do this together now. [Focus group]*

One motivation for going through the testing process together was wishing to know which family member was the gene variant carrier of the disease. Another reason was the family’s wish to support the person affected. In this context, some participants described a certain ‘sense of togetherness’. Assuming the test would have little or no negative consequences, many participants did not see any reasons against undergoing testing together as a family. Some participants refused to test together as a family because, in their view, the test would not change anything. In addition, some participants preferred the state of not knowing: ‘What I don’t know won’t hurt me’ [Focus group]. Although most participants were interested in their family members’ opinions, they also emphasized that the decision for or against testing was up to the patient.

*Everyone else is, of course, asked for their opinion, or perhaps simply what they would do, so that I can hear what they have to say. I want to hear what they have to say, but, at the end of the day, I’m the person who makes the decision. [Focus group]*

### Family planning

The participants described that family planning was an important but difficult issue for them and that genetic aspects mattered. They emphasized the wish to have a healthy child and the concern of passing on the disease.

*It definitely makes the decision more difficult because you’re worried, because you know what could happen. And that’s not very nice and you don’t want that for your children. That’s clear. This will always be in my mind, for years, of course. [Interview 15, Relative]*

Some, especially female, patients reported feelings of fear and guilt of transmitting the disease to their children.

*I felt this between my mother and me and I feel this now between me and my daughter. And, you’re blaming yourself as a mother. You sit there and think, God, I just want the best for my kid, and you give her an illness like that. What kind of mother am I? […] I certainly felt bad about it, some fear, despair and I think my mother had felt the same. [Interview 11, Patient]*

For many participants, the genetic character of the disease was a relevant factor in family planning, particularly in decisions for or against having a child.

*[…] then we were told that the chances that our third child […] will also get the disease is 50/50. ‘It’s your decision’ they said. Then we decided, quite deliberatively, not to have a third child. [Interview 17, Patient]*

In this context, the theme of abortion was discussed and three reasons against having children were raised: firstly, transmitting the illness is a form of harm and it is not acceptable to harm an innocent person like a child. Secondly, it is not acceptable to pass on the burden of disease to a person who cannot be asked and cannot decide against it. Thirdly, to care for an ill child is too burdensome for the family, especially for the mother.

*It’s very hard for me to imagine harming someone else […]. A child can’t say ‘I accept that’ and ‘that’s okay’ and all, but instead the child is born, has the genetic defect and must live with it. […] Because of that, I would say, at the moment, I don’t want children. [Interview 5, Patient]*

Patients and family members reported that the uncertainty whether the disease would be transmitted or not was a burdensome aspect in the decision-making processes for or against having a child. Not knowing whether the child would have the disease led to distress and made the corresponding decision very difficult. In this context, the participants described themselves as powerless.

*That’s like Russian Roulette. [Interview 22, Patient]*

*It’s not nice, but you have no influence. [Interview 15, Relative]*

Some participants stated that they would decide to have children, because they themselves had not experienced the disease as too burdensome and, additionally, that it was not sure whether the child would have the disease. Furthermore, the participants discussed whether it was acceptable to give birth to a child if the expectant mother did not know if she could take care of the child because she did not know how long she would live due to the disease.

*It’s not just the question, does the child have it [the disease], but am I still there as a parent? […] Maybe it’s really a bit selfish to say, yes, I don’t care, I’ll risk it, even if I’m dead in five years, you [the partner] will have to do it alone then, but, yes, I would risk it. [Focus group]*

A few patients indicated that other people, for example, family members, had interfered or tried to influence the decision for or against having children.

*My mother said at that time: You have a boy and a girl and if you know that the disease could come with the third child, what more do you want? You have a boy and a girl. Be satisfied. [Interview 17, Patient]*

## Discussion

Genetic testing during childhood was brought up by the study participants as a major topic and symptoms were seen by the study participants as a major reason for initiating genetic testing. Testing of children who show symptoms has generally been seen as acceptable in literature because it might prevent a long and troublesome period until the correct diagnosis is made. By contrast, predictive genetic testing of children without symptoms is much less acceptable [34–36], particularly regarding incurable diseases, such as hereditary forms of cancer, Alzheimer or Huntington’s disease [18, 37–39]. One problem is that predictive genetic testing in childhood deprives the individual of the opportunity to make an autonomous decision as an adult [27, 36]. The ‘right not to know’ is strongly discussed in this context. Once told, the young person must live with the information about his/her genetic condition. For these (and other) reasons, genetic testing in early childhood is widely rejected [20, 27, 29] which is also mirrored in the present study.

As the discussion in the focus group might suggest, growing up with knowledge about genetic conditions might have effects on the individual’s own health, psychological well-being, self-image, and views about parenthood and family. However, recent literature does not confirm the negative psychological effects of predictive genetic testing [26, 36, 38–41].

Predictive genetic testing can result in exaggerated reactions of the parents, as discussed by the focus group participants. Since parents are often concerned about their child’s further development when a genetic diagnosis is made, early testing can medicalize childhood and, as also described by the study participants, sometimes lead to excessively cautious behaviour [42, 43]. Parents can see their child as ‘at risk’ and treat her/him as vulnerable, for example, restricting physical activities, scrutinizing the child’s development and overusing the medical system [43]. These concerns described in the literature are consistent with the experience expressed in the present study on HCP. Participants reported, for example, that they had been excluded from sports classes in their childhood due to HCP, although—from their own perspective—they would have been able to do sports. Growing up under observation and restrictions can influence the well-being and development of the child and other family members and shape family life in a negative way [43].

The study participants’ argument that there are no reasons for genetic testing in childhood since the test would not change anything is also mirrored in literature regarding other genetic conditions. Professional guidelines on predictive genetic testing of minors usually recommend testing only if effective medical interventions are available to treat, prevent or mitigate the course of a disease [44]. The direct medical benefit to the child is seen as the main justification for predictive genetic testing. If there are no medical consequences, almost all guidelines recommend delaying testing [44]. Since there are, at least currently, no effective interventions or preventive measures for HCP, the IAP also rejects predictive genetic testing for HCP in childhood [29].

As has emerged from the current study, most recommendations suggest delaying testing until the child is old enough to make an informed decision, but there is no consensus about the age at which children can understand the complex issue and give full informed consent [28, 29, 44]. According to the IAP recommendation, a child beyond the age of 12 can begin to contribute to the decision-making process and should, therefore, be included [29]. Many guidelines on predictive genetic testing in minors do not focus on the age itself but instead on the ability of the child to make a free informed decision [44]. The state of development, maturity, competence and understanding are seen as the relevant issues [44]. Participants in the present study named similar conditions to determine the right time for testing. Although different age groups were debated, many participants described adolescence as an appropriate time for testing and rejected testing immediately after birth and in early childhood.

Although professional societies [45] understand genetic testing, in the first place, as an individual and not as a shared choice, participants in the present study described that entire families underwent genetic testing together. Similar to the current study, a previous qualitative study with hereditary pancreatitis patients revealed that the family context plays an important role in decisions regarding genetic testing [46]. Additionally, a systematic review revealed that sharing genetic test results with family members is common [47]. Nevertheless, the review found challenges for the individual in deciding whether to communicate within the family, in assessing what the effects of disclosure could be, in selecting which information to disclose and at what time [47]. Since genetic information does not only affect the individual but also family members, there may be a legitimate interest on the family’s side that relatives decide on testing and share their test results. However, familial relationships and associated responsibilities can affect the choice of the individual in such a way that the free individual choice comes into conflict with the family dynamics [48]. The example in the current study of a parent who tried to interfere in their child’s family planning illustrates this risk and raises the question whether decision-making processes, which involve family members, are appropriate in the context of genetic testing.

Despite longstanding bioethical debates, no agreement has been reached so far on whether and how family members should become part of healthcare decisions [49]. Careful consideration should, thus, be given in the counselling process to the aspect whether the decision for or against a test is made by the individual alone or together with the family and whether the individual wants to share his/her test results. In Germany, for example, any person who is tested must be given individual genetic counselling by a physician before and after predictive genetic testing [50]. Under certain circumstances, the counsellor may recommend that the relatives of the person tested also undergo genetic testing, but the decision to share this information with the family is entirely up to the person tested. Although the counselling process has to cover psychological and social issues regarding the test and its potential results [50], family issues and dynamics should receive more attention.

Participants in the current study also reported that genetic information has influenced or could influence their reproductive behaviour. The use of a prenatal diagnosis for HCP has not yet been investigated, but it could become an issue in the future with the expansion of prenatal testing. The identification of genetic dispositions in the foetus raises difficult questions, for example, about maintaining a pregnancy or not [42, 43, 51]. Because prenatal testing for HCP cannot predict the onset and severity of the condition, the remaining uncertainties make decisions very challenging and can lead to psychological distress for the parents-to-be [43, 51]. Participants in the present study confirmed these concerns by describing the uncertainty of transmitting the disease as a burdensome and stressful dimension in the decision-making process. In addition to psychological problems, difficulties regarding informed consent arise [43, 51]. The expectant parents need unbiased and evidence-based information and support to clarify their own values [51]. A recent review showed that expectant parents have positive attitudes towards learning about the genetic status of their foetuses and choosing among various prenatal testing opportunities, and that they also manage the process very well [51]. Since participants in the current study and those in other studies reported genetic information as an important factor in family planning, accompanied by uncertainties regarding disease transmission, onset and severity of the condition [42, 43, 51], these aspects should be thoroughly addressed in genetic counselling.

## Limitations

Although the current study allows for a deeper understanding of genetic testing in the context of families, the study is subject to the general limitations of qualitative research, such as nonrepresentativeness and subjective interpretations. Since different viewpoints on genetic testing should be covered in the current study, the study also included patients who had decided against genetic testing. Although HCP was therefore not confirmed by genetic testing in every patient, it has been assumed because of both the personal history of pancreatitis and the occurrence of HCP in family members.

Furthermore, the patients’ conditions might have had an influence on the study results: only one of the patients interviewed was in an acute episode at the time of data collection. Talking from a place ‘outside their disease’, the participants might have reported other aspects than in an acute phase. In addition, the study does not have a longitudinal design but, instead, reproduces the participants’ views at a particular point in their lifespan. Longitudinal surveys on HCP patients and their relatives may, in addition, provide further relevant information.

## Conclusion

The current study is the first qualitative study focusing on the experience with genetic testing of HCP patients and their relatives. The study expands previous research on genetic information and, simultaneously, specifies the experience of genetic testing within the context of HCP. The results raise the awareness of the complexity of family contexts: familial relationships, responsibilities and dynamics can have a great influence on decision-making processes. As no agreement has been reached so far on the issues raised in the current study, for example, the right time for genetic testing in childhood or whether and how family members should become part of healthcare decisions, careful consideration should, therefore, be given to these aspects in the counselling process. Increased understanding of the family context can help health professionals to discuss issues related to genetic testing with patients and families better.

## Supplementary information

Supplement 1_Interview guide (version individual patient interview)

Supplement 2_Interview guide (version focus group)

Supplement 3_COREQ-Checklist
