# Supplementary material for: Perceptions of genetic testing in patients with hereditary chronic pancreatitis and their families: a qualitative triangulation
Source: Eur J Hum Genet. 2020 Aug 12;29(1):29–38. doi: 10.1038/s41431-020-00705-9 (PMC7852527; doi:10.1038/s41431-020-00705-9)
Supplement: Supplementary file 1 — Supplement 1_Interview guide (version individual patient interview) [file 41431_2020_705_MOESM1_ESM.docx]

## Interview guide “Ethical aspects in the context of hereditary chronic pancreatitis – a qualitative interview study” (individual interview; patient version)

| **Start/Introduction** | |
| --- | --- |
| Dear [...], I am glad that you are available for an interview with me.  Before we begin, I would like to say a few words about the course of the interview. I would like to record the interview so that I could focus more on talking with you. Do you agree?  [If the participant agrees, switch on the recorder. If the participant does not agree, take notes.]  The recorder is running now. We have already talked about the voluntariness of your participation in this research project. Please feel free to interrupt or to end the interview at any time you want. Otherwise, you can talk as much as you like – I have time.  Do you have any questions about the interview?  Today, I would like to talk about your disease, hereditary chronic pancreatitis.  How did you realize that you have this disease? | Introduction  Recorder  Voluntariness  Interruptions  Encouragement  Questions  Start |

| **Theme I: Patient biography** | |
| --- | --- |
| The diagnosis is often a long process. Would you tell me something about it?  How did you realize you were ill?  How/when did you hear that you have pancreatitis?  Has something changed since the diagnosis?  What happened after diagnosis?  What is it like to live with the disease? (Changes between “normal” and “acute” illness phases?)  How are you doing with the disease right now?  Do you have any restrictions in your daily life?  Does the disease affect your education/job?  Does the disease affect your family life?  Would you complete the following sentence for me:  Living with chronic pancreatitis means for me ... | Way to diagnosis  Living with the disease  Education/school/job  Family life |

| **Theme II: Genetic testing** | |
| --- | --- |
| We have already talked about the long way to diagnosis. Today, it’s also possible to carry out a genetic test. Have you done such a test?  *Yes/No*: Can you tell me, why did you decide in this way?  Would you describe for me which aspects influenced your decision?  Can you pretend to be in this situation again? How was that? Would you decide differently today?  Was there any consultation? If so, how did you feel about it?  Did you include other people in the decision-making?  *If participant did the test:*  What did you do with the information?  Did you share the information with other people? If so, how did they react?  Has something changed in your life because of the information?  Did the information affect something? Can you give me an example?  So far, we have talked a lot about chronic pancreatitis; does the topic "cancer" also play a role in your life?  What do you think of when I bring up the topic "cancer"?  How do you deal with this issue? | Motivation  Decision making process  Consultation  Family  Dealing with the information  Influence of information  Cancer |

| **Theme III: Patient self-help groups** | |
| --- | --- |
| We have contacted you about the association “XX". Can you tell me how you came into contact with this group? / Why did you become a member of this group?  Would you tell me what this group does?  What are you talking about in the group? Does the group also invite external people?  How do you help each other?  Is there anything else you want to tell about the group?  Can you describe an example of how you get support from the group?  Are you missing something?  As a patient, do you want more support, for example, from your doctor? | Reasons  Functions  Role  Support |

| **Theme IV: Research participation** | |
| --- | --- |
| As a last point, I would like to talk to you about the topic "biomedical research."  Have you ever participated in a clinical study?  *Yes:* Can you tell me how it came about? Why did you participate in the study?  (What kind of study? What did you do? Do you know the results of the study?)  *No: Two possibilities: Patient has not yet had the opportunity to participate in a study or patient has decided against participation.*  Imagine that biomedical research had enough money and all the technical instruments to explore anything you wanted. Regarding chronic pancreatitis, what would you like explored? | Experience  Motivation  Reasons against  Wishes |

| **Open ended** |  |
| --- | --- |
| Do you want to tell me something that we have forgotten to speak about in the interview?  Is there anything else you want to add? |  |
| **Social demographics** | |
| Finally, I have only a few more specific questions… | |

**Thank you very much!**
