# Supplementary material for: Perceptions of genetic testing in patients with hereditary chronic pancreatitis and their families: a qualitative triangulation
Source: Eur J Hum Genet. 2020 Aug 12;29(1):29–38. doi: 10.1038/s41431-020-00705-9 (PMC7852527; doi:10.1038/s41431-020-00705-9)
Supplement: Supplementary file 2 — Supplement 2_Interview guide (version focus group) [file 41431_2020_705_MOESM2_ESM.docx]

# Interview guide: ‘Ethical aspects in the context of hereditary chronic pancreatitis – focus group’

| Start | |
| --- | --- |
| Introduction of the moderator, the assistance and their tasks  Expressing thanks  Information about the study | My role is that of a moderator: I will ask you some questions and control a little bit the discussion.  However, you can talk freely to each other.  My assistance keeps an eye on the time and writes a small protocol.  I am glad that you participate in this focus group session.  Some of you know our study already, but not everyone knows what our project is about. In our study we want to investigate how it is to live with Hereditary Chronic Pancreatitis (HCP). We are interested in your experience with research, genetic testing and patient self-help groups. To obtain the views of patients and relatives on these topics, we have already conducted interviews. Today, we want to complement these interviews with a group discussion and I am looking forward to discuss the topics with you. |
| Recorder | Before we begin, I would like to say a few words about the course of the discussion. I would like to record the discussion so that I could focus more on talking with you. Do you all agree?  [If the participants agree, switch on the recorder. If the participants do not agree, take notes.]  The recorder is running now. |
| Voluntariness  Interruptions  Encouragement  Confidentiality  Questions | We have already talked about the voluntariness of your participation in this research project. Please feel free to interrupt or to end the group session at any time you want. Otherwise, you can talk as much as you like – We have time.  Please keep the statements that will be made within this group to you. This is important to build a trustful atmosphere for the discussion.  If there is anything that you do not want to say in front of the group, you can write it down and tell me later, for example after the group session.  As I have already explained, I have the role of a moderator. But, I will lead the conversation just a little bit; you can talk freely to each other.  There is only one rule: Please do not interrupt each other. Otherwise, you can talk as much and freely as you like.  Are there any questions about the discussion? |
| Introduction of the participants | Some of you already know each other. Nevertheless, I would like to ask you to write down your names on the cards, introduce yourself in one or two sentences and explain why you are taking part in this patient day for HCP. |

| Family life | |
| --- | --- |
| Family life  Input: Quotes Consequences of HCP | I'd like to start with the topic of 'family life', because we found very different formulations in the interviews. I brought you two quotes as examples.  One participant told us: ‘The family comes close because of the disease.’  But another participant said: ‘The family broke because of the disease.’  You can take one of the cards and say something about it, if you want.  Can you understand one or both of these formulations? |

| Family planning | |
| --- | --- |
| Family planning  Decision for or against children  Reasons  Deciding under uncertainty  Feelings of guilt | I would like to discuss another point with you about 'family life'. Some participants in the interviews told us that the genetic character of HCP had an influence on their decision for or against children.  I know this is a very difficult topic. Just say something about it, if you want.  Would you tell me your experience / your thoughts?  Is there anything else you want to tell about this topic?  In this context, one participant said: ‘It's like Russian roulette.’  Can you explain that to me?  Another participant told us: ‘[...] you're blaming yourself as a mother. You sit there and think, God, I just want the best for my kid, and you give her an illness like that. What kind of mother am I?’  Can you understand that?  How do you deal with this issue? |

| Genetic testing within the family | |
| --- | --- |
| Genetic testing  Counselling  Testing with the family together  Input: Role cards  Who should decide?  Time of testing  Input: Timeline  Testing in childhood | As patients you have already undergone various tests. Today it is possible to do genetic testing for HCP. Is genetic testing different from other tests, such as a blood test, in your view?  Would you tell me what the difference is?  Did you have any counselling before or after the testing?  How did you feel about the counselling?  Some participants told us in the interviews that the genetic test was underwent by the family together.  How was it in your case?  How do you feel about it?  Hand out the cards (doctor, patient, partner, children, parents, friend and plain cards). Who should participate in the decision for or against testing and why?  Would you like to say something about these roles?  We have already talked about who should decide or be involved in the decision-making process, but when do you think is the right time for genetic testing? You can take a pen, put a cross on the timeline and say something about it, if you want.  Some participants in the interviews reported genetic testing in childhood.  What do you think about that? |

| Open ended |  |
| --- | --- |
| Do you want to tell something that we have forgotten to speak about it in the group?  Is there anything else you want to add? |  |
| Social demographics | |
| Finally, I have only a few more specific questions… | |

**Thank you very much!**
